# Can Mesoporous Silica Speed Up Degradation of Benzodiazepines? Hints from Quantum Mechanical Investigations

**DOI:** 10.3390/ma15041357

**Published:** 2022-02-12

**Authors:** Massimo Delle Piane, Marta Corno

**Affiliations:** 1 Department of Applied Science and Technology (DISAT), Politecnico di Torino, Corso Duca degli Abruzzi, 24, 10129 Torino, Italy; massimo.dellepiane@polito.it; 2 Dipartimento di Chimica and Nanostructured Interfaces and Surfaces (NIS) Centre, Università degli Studi di Torino, Via Pietro Giuria, 7, 10125 Torino, Italy

**Keywords:** mesoporous silica, DFT, nitrazepam, drug delivery systems

## Abstract

This work reports for the first time a quantum mechanical study of the interactions of a model benzodiazepine drug, i.e., nitrazepam, with various models of amorphous silica surfaces, differing in structural and interface properties. The interest in these systems is related to the use of mesoporous silica as carrier in drug delivery. The adopted computational procedure has been chosen to investigate whether silica–drug interactions favor the drug degradation mechanism or not, hindering the beneficial pharmaceutical effect. Computed structural, energetics, and vibrational properties represent a relevant comparison for future experiments. Our simulations demonstrate that adsorption of nitrazepam on amorphous silica is a strongly exothermic process in which a partial proton transfer from the surface to the drug is observed, highlighting a possible catalytic role of silica in the degradation reaction of benzodiazepines.

## 1. Introduction

In order to be easily administered, delivered to the target organs and stabilized, a molecule with a pharmacological activity needs to be accompanied by many other different chemical entities. Usually defined as “pharmacologically inactive”, they play an essential role ensuring the correct functionality of a medicament. The whole system is defined as “the dosage form of a drug” and traditionally consists of the active pharmaceutical ingredient (API), which is the drug itself, that is the biologically active molecule in the organism and is at the base of the therapeutic value of the medicament and the excipients, a heterogeneous group of non-therapeutic substances, which are intended to attribute desirable and practical properties to the dosage form [1,2,3].

During most of the 20th century, excipients have been generally considered as substances added to the final formulation of a drug in order to obtain the weight, volume, and consistency necessary for the correct administration of the active principle to the patient [1]. However, the common definition of excipients as “inert” or “inactive” is now considered inaccurate since they often possess reactive functional groups and/or belong to classes of compounds that can modify the physical properties of the mixture they form. A few studies have underlined in the past how the carrier can speed up the degradation of the active principle contained. Excipients and impurities they can carry may directly interact with active ingredients by participating in chemical reactions by acting as catalyzers and/or by adsorbing drugs through hydrogen bonding and other non-covalent interactions [2,4,5,6].

Some excipients are capable of adsorbing active ingredients to their surfaces [3,7,8]. This process can be driven by the establishment of interactions of different strength, such as Coulomb forces, hydrogen bonds, and van der Waals forces (dipole–dipole interactions, dipole-induced dipole interactions, and London forces). The number and kind of interactions between drugs and excipients cooperatively determine the binding energy of the adsorbate [3]. Moreover, effects on drug activity and stability are dependent on the geometry of adsorption and on the part of the molecule implicated in the interaction. The adsorption of a molecule on a surface is the first step of every reaction catalyzed by the surface. However, the adsorption process of drugs on excipients is not only important for its possible role in catalyzing degradation reactions. The formation of strong interactions between the active ingredient and other component of the formulation can strongly affect its dissolution rate and thus significantly alter absorption and bioavailability [2,9,10]. Adsorption of active ingredients on excipients can be carefully exploited to optimize the dissolution rate of the product, in the so-called controlled drug release [3,11,12,13]. This approach might be extremely useful to obtain a reduction in daily administration of drugs with fast absorption and/or elimination. It is used also to develop sustained release dosage forms that can maintain a fixed drug concentration in body fluids for a specific period of time [13,14].

Amorphous silica is a key material with a variety of applications in many fields such as chromatography, microelectronics, and metal-supported catalysis [15,16]. It has been commonly used as a solid additive in pharmaceutical dosage forms primarily as a tableting excipient, facilitating the manufacturing and durability of tableted products. This usage stems essentially from its anticaking capability, that is the capacity, when added to powered or granulated materials, to prevent the formation of lumps and to increase the free-flow of tableting powders [3]. When added to a marketed product, silicon dioxide is usually listed among the ingredients as E551 [17]. The interest in pharmaceutical employment of amorphous silica has rapidly grown in recent years following the development of silica-based mesoporous ordered materials and the discovery of their biomedical applications [11,18,19]. Mesoporous materials are synthesized using supramolecular assemblies of surfactants as templates for inorganic components, commonly silica. After removal of the surfactant, the resulting mesoporous matrices exhibit some striking features, such as an ordered pore network with high homogeneity in size and very high pore volume and surface area. The unique features of mesoporous silica make it an excellent candidate for drug delivery systems [20,21,22,23]. Since 2001, when MCM-41 was firstly proposed as a drug delivery carrier [11], many silica-based mesoporous materials have been discussed as possible drug carriers [22].

**Figure 1:**
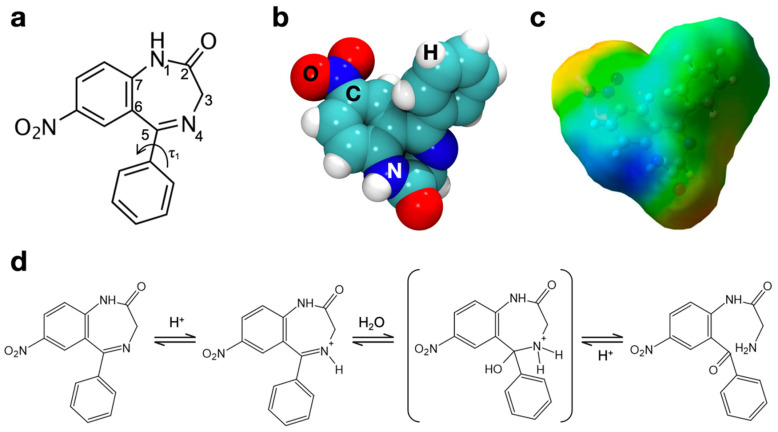
(**a**) 2D chemical structure of nitrazepam with the canonical numbering. (**b**) 3D space filling model of the most stable conformer of nitrazepam. (**c**) Electrostatic potential map of the most stable conformer of nitrazepam. Blue regions correspond to positive values of the potential, green to neutral and red to negative potential. Potential range: MIN: −0.02 e – MAX: +0.02 e. 3D ball and stick structure of the molecule is shown under the potential surface. (**d**) The ring-opening reaction of 1,4-benzodiazepines for the specific case of nitrazepam.

Benzodiazepines are psychoactive drugs whose core structure is based on the fusion between a benzene ring and a diazepine ring, that is a seven-member heterocycle with two nitrogen atoms located in Positions 1 and 4 (1,4-benzodiazepines) [24,25,26]. They bind to GABA (γ-aminobutyric acid) receptors in the central nervous system, enhancing the binding of this neurotransmitter and thus increasing its biological action [27]. Since GABA is a major inhibitory neurotransmitter, benzodiazepines have a sedative, hypnotic, anxiolytic, and anticonvulsant action. Nitrazepam (1,3-dihydro-7-nitro-5-phenyl-2H-1,4-benzodiazepin-2-one, C_16_H_11_N_3_O_3_) is a 1,4-benzodiazepine used for the treatment of severe insomnia [28]. Nitrazepam’s diazepine ring contains, like many other benzodiazepines, an azomethine group with the double bond between N4 and C5 (Figure 1a,b). This Schiff-base bond (R2C=NR) is hydrolysable in aqueous solutions under acid catalysis. This ring opening reaction (Figure 1d), which leaves an aldehyde and a primary amine on the two free terminals, is the first step in nitrazepam degradation in an aqueous environment [26,29]. However, since it is a reversible reaction, some other benzodiazepines exist as an equilibrium of the open and the closed form in solution at standard conditions [29].

Given the broad use of silica as a pharmaceutical excipient, it is therefore of paramount importance to understand whether its surface can have a catalytic role in the hydrolysis of 1,4-benzodiazepines and of nitrazepam as a test case. The kinetics of this degradation reaction by silica were indeed studied in 1982 [30]. It was hypothesized that adsorption of this drug on silica, through hydrogen bonding with superficial silanols, could be the mechanism of this catalysis. In particular, since the first step of the hydrolysis of this compound proceeds by opening of the azomethine group, even weak adsorptive bonds should alter the electron density in the vicinity of the nitrogen atom and facilitate the attack by water. The silanol groups could therefore act as weak acid catalyzers. However, although a possible adsorption geometry was proposed on the basis of IR spectra [30], the precise mechanism of this interaction is still not known at a molecular level.

The present study reports a quantum mechanical modeling of nitrazepam in interaction with amorphous silica surfaces, as models for the walls of silica mesopores. This approximation proved valid in a previous study on clotrimazole encapsulation in mesoporous silica [13]. Our modeling follows the same approach already considered when studying adsorption models of both aspirin and ibuprofen drugs [3,7,20], extended to consider the dynamics and possible reactivity of the silica–nitrazepam system. The aim of the present study is to investigate the role of silica surfaces in the interaction with nitrazepam to state if there could be an effect on the drug availability when considering silica as the carrier in a pharmaceutical formulation.

## 2. Computational Details

### 2.1. Static Quantum Mechanical Calculations

All the static calculations were performed within the density functional theory (DFT) with the CRYSTAL17 code [31] in its massively parallel version [32]. The chosen functional was the Perdew, Burke, and Ernzerhof GGA (Generalized Gradient Approximation) exchange-correlation functional (PBE) [33], with and without including the empirical Grimme’s D2 correction [34], to describe the dispersive interactions (vdW). In the following, the notations PBE-D2 and PBE will be used to determine whether the Grimme’s correction is included or not, respectively. Split valence double- (for Si atoms) and triple-ζ (for other atoms) Gaussian type basis sets plus polarization functions were used to describe the systems [35,36]. Only the atomic coordinates of the two more superficial layers of each silica slab in the docking geometries were optimized, to compensate for the reduced thickness of the models.

Interaction energies, per unit cell per adsorbate molecule (∆E), were calculated and corrected for the basis set superposition error (BSSE) according to the counter-poise methodology as used in previous papers by Delle Piane et al. [3,13,20]. The interaction energy, ΔE, per unit cell per adsorbate molecule is a negative quantity (for a bounded system) defined here as:

ΔE = E(SN//SN) − [E_M_(N//N) + E(S//S)] (1)

where E(SN//SN) is the energy of a fully relaxed silica model S in interaction with the nitrazepam drug molecule N, E(S//S) is the energy of a fully relaxed silica model alone, and E_M_(N//N) is the molecular energy of the free fully optimized ibuprofen molecule (the symbol following the // identifies the geometry at which the energy has been computed). The energy of deformation due to the change in geometry of both the drug and the material upon interaction can be taken into account by means of the following expressions:

ΔE = ΔE* + δE_S_ + δE_N_ (2)

δE_S_ = E(S//SN) − E(S//S) (3)

δE_N_ = E(N//SN) − E_M_(N//N) (4)

ΔE* = E(SN//SN) − [E(N//SN) + E(S//SN)](5)

in which δE_S_ is the deformation energy of the silica surface, whereas δE_N_ (ΔE_N_ + ΔE_L_) counts both the deformation energy of the nitrazepam molecule (ΔE_N_) and the lateral intermolecular interactions (ΔE_L_) between the periodic replicas of the same nitrazepam molecule along the *c* direction, i.e., along the pore length. The purely molecule deformation energy can be computed as:

ΔE_N_ = E(N//SN) − E_M_(N//N) (6)

in which E(N//SN) is the molecular energy of the molecule frozen at the geometry occurring on the silica surface. The lateral intermolecular interactions, ΔE_L_, are defined as:

ΔE_L_ = E(N//SN) − E_M_(N//SN) (7)

and can be either positive (repulsion) or negative (attraction). The ΔE* interaction energy is then deformation and lateral interactions free. The above ΔE definition can be easily reformulated to include the basis set superposition error (BSSE) correction, using the same counterpoise method adopted for intermolecular complexes [37]. The definition of the BSSE corrected interaction energy ΔE^C^ is then:

ΔE^C =^ ΔE*^C^ + δE_S_ + ΔE_N_ + ΔE_L_ (8)

ΔE*^C^ = E(SN//SN) − [E(S[N]//SN) + E([S]N//SN] (9)

BSSE = ΔE^C^ − ΔE (10)

in which E(S[N]//SN) and E([S]N//SN) are the energy of silica plus the ghost functions of the drug molecule and the energy of the infinite replica of molecules with the ghost functions of the underneath silica framework, respectively.

### 2.2. Frequency Calculations

Harmonic frequencies were calculated with CRYSTAL17 at Γ point and the infrared intensity for each normal mode was obtained by computing the dipole moment variation along the normal mode, adopting the Berry phase method [38]. For the simulation of the IR spectra of the different structures, only a fragment consisting of the adsorbate has been considered for constructing the Hessian matrix.

### 2.3. Ab Initio Molecular Dynamics

Ab-initio molecular dynamics (AIMD) simulations were performed using the CP2K code. [39] The Quickstep technique [40] with a mixed plane wave and Gaussian basis set methodology (Gaussian and Plane Wave method, GPW) was employed to calculate the electronic structure. We used the PBE functional, with the Goedecker−Teter−Hutter pseudopotentials [41] and a triple-ζ basis set with polarization functions (TZVP) [42] augmented with the empirical Grimme’s D2 correction [34]. The cutoff for the plane wave basis was set to 400 Ry. AIMD simulations were run at 300 K in the NVT ensemble, using the Canonical Sampling through Velocity Rescaling (CSVR) thermostat [43]. A time step of 0.5 fs was chosen. All simulations were equilibrated at 300 K with a more stringent thermostat (time constant: 10 fs) for about 1 ps and then the production phase was run for 12 ps with a more relaxed thermostat (time constant: 50 fs). Since CP2K requires 3D periodic systems, a value of c = 25 Å was chosen to separate the slab replicas with enough vacuum.

## 3. Results and Discussion

### 3.1. Amorphous Silica Surfaces

Surface chemistry of amorphous silica is mainly based on the concentration and activity of superficial silanol groups (-SiOH) [8,16,44,45]. These functionalities may exist in a variety of conditions, and they may be either isolated or interacting with each other through hydrogen bonding as a function of thermal treatment. Silica samples treated at increasing temperature undergo a dehydroxylation process, resulting in more hydrophobic surfaces: this happens via condensation of silanols, which are close enough to interact, with elimination of a water molecule and formation of a siloxane bond [8,21].

**Figure 2:**
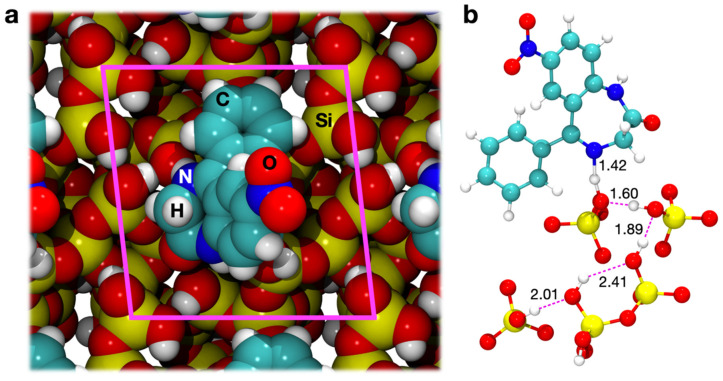
(**a**) Space filling top view of the PBE-D optimized adsorption geometry of nitrazepam on the 4.5 OH/nm^2^ silica surface. Unit cell borders are indicated in magenta. (**b**) Hydrogen bonds between nitrazepam and the exposed silanols in the PBE-D optimized adsorption geometry. Bond lengths are in units of Å.

**Figure 3:**
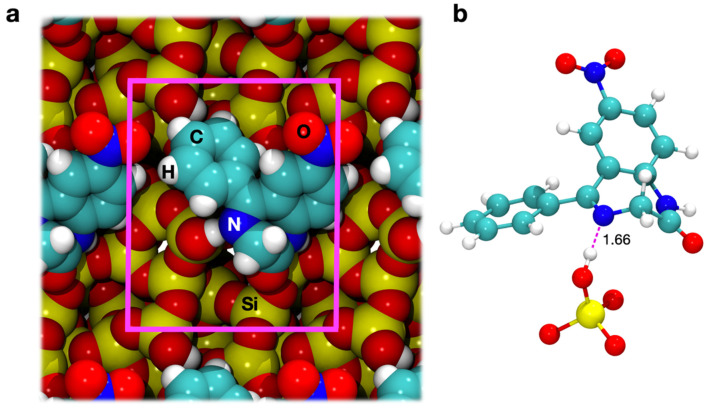
(**a**) Space filling top view of the PBE-D optimized adsorption geometry of nitrazepam on the 1.5 OH/nm^2^ silica surface. Unit cell borders are indicated in magenta. (**b**) Hydrogen bonds between nitrazepam and the exposed silanols in the PBE-D optimized adsorption geometry. Bond lengths are in units of Å.

With this in mind, our group has previously designed amorphous surfaces with different densities of surface silanols sporting various degrees of hydrophilicity. [46] For this paper, two surfaces derived from an amorphous bulk were adopted from that work (visible below nitrazepam in Figure 2 and Figure 3): a fully hydroxylated surface (4.5 OH/nm^2^) and a much more hydrophobic one (1.5 OH/nm^2^), already employed to simulate the adsorption of ibuprofen and aspirin [3]. The use of flat surfaces in the present work, at variance with an explicit model of mesoporous silica, is justified by the curvature of the mesopores, whose average diameter (5–10 nm) is generally much larger than the drug molecule so that a flat surface model represents a very good approximation of what the drug is approaching inside the pores.

In the chosen 4.5 OH/nm^2^ surface, the high number of silanols, whose density is close to the experimentally measured value for fully hydroxylated surfaces (4.9 OH/nm^2^) [45], is mirrored in the high number of hydrogen bonds. Out of the eight silanols in the unit cell, only one is non-interacting. One silanol is acting both as an acceptor and a donor of hydrogen bond and, together with two other SiOHs, is cooperating in forming a chain of interactions: it is known that, in this kind of chain, the proton of the terminal hydroxyl is more acidic than average [3,13], and this feature played a significant role in the adsorption mechanisms tested in this paper. Finally, one geminal silanol can be observed. For the 1.5 OH/nm^2^ surface, silanol groups are all non-interacting, and one group is partly buried inside the surface, mostly excluded from possible interactions with incoming molecules [3].

### 3.2. Nitrazepam

The benzodiazepine nitrazepam is a more complex molecule than the drugs considered in our previous papers on the topic, aspirin and ibuprofen [3,20]. Although it has almost the same number of atoms than ibuprofen (32), its chemical structure, represented in Figure 1a, is far more complex. The 7-atom diazepine ring is condensed with a nitro-phenyl group, and it is substituted in Position 5 by another phenyl ring and in Position 2 by a carbonyl group. Moreover, this functional group belongs to an amidic bond comprising N1. The functional complexity is expected to give rise, when nitrazepam is adsorbed on amorphous silica, to a large number of adsorption geometries with similar energy.

The molecular structure was downloaded from PubChem database [47] and optimized in gas phase at the chosen level of theory (Figure 1b). It was then verified that geometry optimization converged on the most stable conformer by comparison with previous works in the literature: a recent ab initio conformational analysis [48] confirmed that the obtained structure corresponded to the most stable conformer of this molecule. The molecule was also optimized without dispersion to be coherent with adsorption geometries obtained without this contribution: no significant difference in the resulting structure was noticed.

The electrostatic potential of this molecule was mapped on the DFT electron density and is represented in Figure 1c. This molecule appears more polar than aspirin and ibuprofen [3]. Although the phenyl substituent is definitely apolar and is a candidate for strong dispersive interactions with the surface, the rest of the molecule exposes a series of polar functionalities. A negative region surrounds the nitro group, while for the amide group, the different polarity of the NH portion (positive) and of the carbonyl (negative) is clearly seen. Furthermore, the nitrogen atom involved in the Schiff base bond of the diazepine ring is characterized by a slightly negative potential. The electrostatic potential map of Figure 1c therefore shows that multiple functional groups of nitrazepam are able to interact with surface silanols, and this must be taken into consideration when studying its adsorption.

### 3.3. Nitrazepam Adsorption on Amorpohous Silica Surfaces

#### 3.3.1. Geometry Optimization

The azomethine group of the diazepine ring is a Schiff base bond between N4 and N5 (see Figure 1a), which is hydrolysable in aqueous solutions under acid catalysis (Figure 1d). As stated in the Introduction, this ring opening reaction is the first step in nitrazepam degradation in aqueous environment [26,30,49]. The relationship between nitrazepam degradation and adsorption on amorphous silica has been experimentally studied by Czaja and colleagues several years ago, by interpreting infrared spectra of nitrazepam on a silica surface with a silanol density of 3 OH/nm^2^ [30]. They suggested an adsorption geometry in which all polar functional groups of nitrazepam make contact with the surface silanols. When taken into practice, this geometry of adsorption was found to be impossible, because the conformation of the drug prevents the concurrent interaction of all the polar groups of the molecule, at least on a silica surface with less than 5 OH/nm^2^. The critical interaction in influencing the degradation kinetics of nitrazepam, however, is supposed to be the hydrogen bond between the nitrogen atom of the azomethine group (N4, Figure 1a) and surface silanols: this interaction can alter the electron density of the molecule, weakening the C=N bond and increasing the rate of its hydrolysis. To simulate the docking of nitrazepam on our silica surface models, the starting geometries were generated, trying to maximize the interaction of the nitrogen atom of the Schiff base bond.

The PBE-D2 optimized geometry of adsorption on the surface with a silanol density of 4.5 OH/nm^2^ is shown in Figure 2a, through space-filling models. As was already seen for aspirin and ibuprofen [3], we observed that the inclusion of dispersion moves the molecule closer to the surface, driven by the vdW interactions of the aromatic part of the drug. A significant deformation of the surface (δE_S_) was computed for both PBE and PBE-D cases, due to rearrangements of the surface silanols to maximize the interaction with N4. Indeed, as Figure 2b highlights, this nitrogen is an acceptor of a H bond from a silanol that is terminal of a 5-membered chain. This chain of interacting silanols was shorter on the bare surface, indicating that the silanols have undergone a profound rearrangement in response to drug adsorption. This phenomenon had already been observed for ibuprofen adsorption on the pore walls of mesoporous silica [20]. This interaction causes an incipient proton transfer between the terminal silanol of a surface chain toward the N4 nitrogen of nitrazepam, driven by the strong acidity of this surface moiety. The transfer is further emphasized by dispersion interactions, which bring the nitrazepam closer to the surface. This proton transfer may be described as the first step in the catalytic opening of the diazepine ring (Figure 1d). No significant interactions are observed for the other polar functional groups of the drug.

Additionally, in the case of the surface with a silanol density of 1.5 OH/nm^2^, the starting adsorption geometry was obtained by maximizing the interactions between the nitrogen atom of the azomethine group and the only exposed silanol of the cell. The position of nitrazepam on the surface is quite different from the hydrophilic case (Figure 3a). Now, the molecule lies on the surface without portions protruding far away from the surface. A local view of the H-bond interaction is provided by Figure 3b. The H---O bond is longer than that computed for the hydrophilic surface (see Figure 2b). This was expected as for the hydrophilic surface the H-bond is with a silanol terminal of a rather long H-bonded chain of surface silanols. Interestingly, however, the length of this interaction lies in the lower range of those measured for other drugs on amorphous silica [3,13,20], suggesting that even an isolated silanol on an hydrophobic surface can potentially weaken the neighboring C=N bond.

#### 3.3.2. Energetics

In our previous investigations [3,20], we stressed the significance of energies computed by including/excluding the dispersive interactions in considering the adsorption energies for the adsorption geometries. Indeed, when excluding such contributions, the only driving force for the nitrazepam−silica interaction is the formation of H-bonds between silanols and the diazepine ring of the molecule. The rest of the drug is affected by steric repulsion with respect to the underlying surface, resulting in a balance of these two effects. Including dispersion interactions, via the Grimme’s empirical correction, dramatically changes the above scenario by opposing this pure electronic repulsion and becoming a significant fraction of the interaction energy. Separating these contributions is therefore of relevance, particularly since it has been found that dispersive interactions are not merely additive to existing H-bonding ones, but a competition exists between the two interactions, with important structural and energetic consequences for the adsorption [3]. Table 1 provides all the calculated interaction energies for adsorption of nitrazepam on the hydrophilic and hydrophobic silica surfaces, at both the PBE and PBE-D2 levels of theory.

Considering the 4.5 OH/nm^2^ surface, the PBE interaction energy is −30.8 kJ·mol^−1^, comprising the energy of the only H-bond between the molecule and the surface present in this geometry. This dramatically becomes −104.9 kJ·mol^−1^ when dispersion is included, so that the total interaction energy of this system is by more than 90% constituted by pure dispersion. As was the case for ibuprofen [3], dispersion constitutes the main driving force of nitrazepam adsorption, and it is thus evident that simulating such systems by means of standard DFT functionals lacking the inclusion of dispersions would result in a poor description of their energetics.

As regards the 1.5 OH/nm^2^ surface, the ∆E^D^, that is, including dispersion, is computed as −131.9 kJ·mol^−1^, which is higher than the ∆E^D^ for the hydrophilic case. This is at variance with what was observed for aspirin and ibuprofen on the same amorphous silica surface, where interaction energies on the 1.5 OH/nm^2^ surface were computed as significantly lower than the ones for the 4.5 OH/nm^2^ surface [3]. This difference may be due to the high deformation of the surface observed for the hydrophilic case in interaction with nitrazepam, despite a shorter H-bond and an incipient proton transfer, compared to the longer H-bond in the hydrophobic case. Furthermore, it proves how hard it is to predict a priori the adsorption strength of drugs on amorphous silica, with many contributions in a delicate and interconnected balance. As for the hydrophilic case, this energy is mainly constituted by dispersion, which alone represents 79% of the total energy. Finally, if the ∆E, i.e., without including dispersion, can be considered a good estimate of the energy of the H-bond interaction, this value is comparable to what observed on the hydrophilic surface for the PBE model (−38.7 vs. −30.8 kJ·mol^−1^).

Finally, a similar competition between directional H-bonds and nonspecific dispersion-driven interactions as observed for other drugs [3] is found here, as proven by the purely PBE interaction energies (∆E) computed on structures optimized at the PBE-D2 levels of theory. These are indeed smaller that the PBE optimized cases (−10.1 vs. −30.8 kJ·mol^−1^ for the 4.5 OH/nm^2^ surface and −28.2 vs. −38.7 kJ·mol^−1^ for the 1.5 OH/nm^2^ surface), while, if dispersion had no competing effect in determining adsorption, these two values would have been very similar. As already stated above, dispersion contribution is therefore not simply additive to other energy terms. Surprisingly, however, this effect is stronger on the hydrophilic case, where one would expect a smaller role of dispersive contributions.

#### 3.3.3. Molecular Dynamics

So far, we have provided only a static picture of nitrazepam molecules adsorbed on silica surfaces, as models of mesoporous silica pore walls. These simulations provide only a partial picture of what happens in the actual system. Indeed, they do not offer relevant information regarding (i) the mobility of the drugs after adsorption at the surface and how stable these configurations are or (ii) the dynamicity of the proton transfer process that we suggest as the first step in the silica-induced degradation of nitrazepam. The first point is also quite relevant, since some experimental results (particularly solid-state NMR) have suggested that ibuprofen incorporated in mesoporous silica shows a very high mobility, almost “liquid-like” [50,51]. Such a mobility may also be shared by nitrazepam loaded in mesoporous silica. To explore the above-mentioned points, a different approach from the static calculations described in the previous sections is required. For this reason, we report here the results of Ab Initio Molecular Dynamics (AIMD) simulations performed at room temperature (300 K) on the static adsorption structures described above. These AIMD simulations have been carried out using the same PBE-D2 functional employed for static calculations.

**Figure 4:**
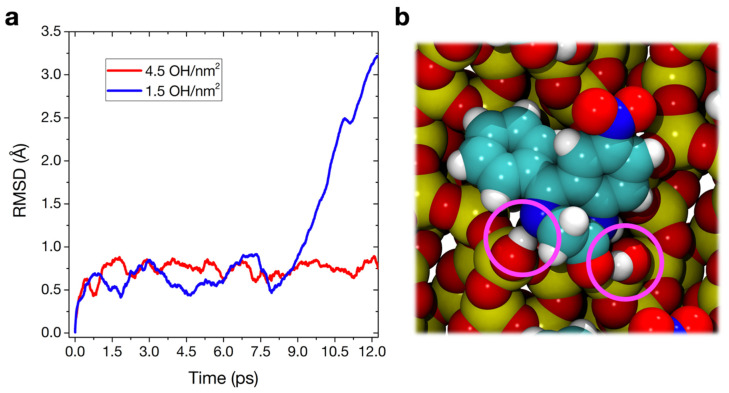
(**a**) Root mean square deviation of the atomic positions (in Å) with respect to the initial structure along the AIMD trajectories, for nitrazepam adsorbed on the 4.5 (red) and 1.5 (blue) OH/nm^2^ silica surfaces. (**b**) Space filling top view of the PBE-D optimized adsorption geometry of nitrazepam on the 1.5 OH/nm^2^ silica surface, resulting after 12 ps of AIMD starting from the structure of Figure 3.

In MD simulations, one common measure of the mobility of the model during the simulation is the root–mean–square deviation (RMSD) of the atomic positions:

(11) $$RMSD=\sqrt{\frac{1}{N}\overset{N}{\underset{i=1}{\sum }}\delta_{i}^{2}}$$

where *δ* is the distance between *N* pairs of equivalent atoms. In Figure 4a, the *RMSD* is calculated between each MD step and the starting geometry, for both nitrazepam adsorbed on the 4.5 and 1.5 OH/nm^2^ silica surfaces. The graph demonstrates that, while the adsorption geometry in the hydrophilic case is equilibrated during the whole simulation, with little changes in structure, the nitrazepam molecule on the hydrophobic surface is extremely mobile, and its movement can be traced according to the variation of its RMDS in time. A starting position is kept during the first 8 ps; then, the drug performs a large movement between 8 and 12 ps that results in a new configuration (Figure 4b), with an additional H-bond with the carbonyl group of nitrazepam. The RMSD after 12 ps of MD is 3.2 Å with respect to the starting point for the 1.5 OH/nm^2^ case and just 0.7 Å for the 4.5 OH/nm^2^ case. This difference in stability at room temperature is remarkable, since static calculations predicted a stronger interaction energy for the hydrophobic surface case. However, both the stability of the long H-bonded silanol chain and the proton transfer reaction at the N4 of nitrazepam clearly result in much less room available for structural flexibility at 300 K. More translational and rotational freedom is allowed, on the other hand, by the single H-bond with an isolated silanol present on the 1.5 OH/nm^2^ surface.

We took the new geometries obtained after 12 ps and optimized them statically at the PBE-D2 level of theory. We then computed the corresponding interaction energies (∆E^D^), reported in Table 1. While very limited geometrical changes happen during the AIMD for the 4.5 OH/nm^2^ case (Figure 4a), the little rearrangements of silanols and drug, allowed by the added kinetic energy, result in a more stable adsorption geometry (−104.9 vs. −128.0 kJ·mol^−1^ before and after AIMD, respectively). The newly found adsorption geometry on the 1.5 OH/nm^2^ surface (Figure 4b), although sporting one additional H-bond with respect to the one of Figure 3, has a computed ∆E^D^ of −100.2 kJ·mol^−1^, lower than before (−131.9 kJ·mol^−1^): the two H-bonds compete with each other and the silanol–N4 interaction, the driving force of the original adsorption, is destabilized as a consequence. 

**Figure 5:**
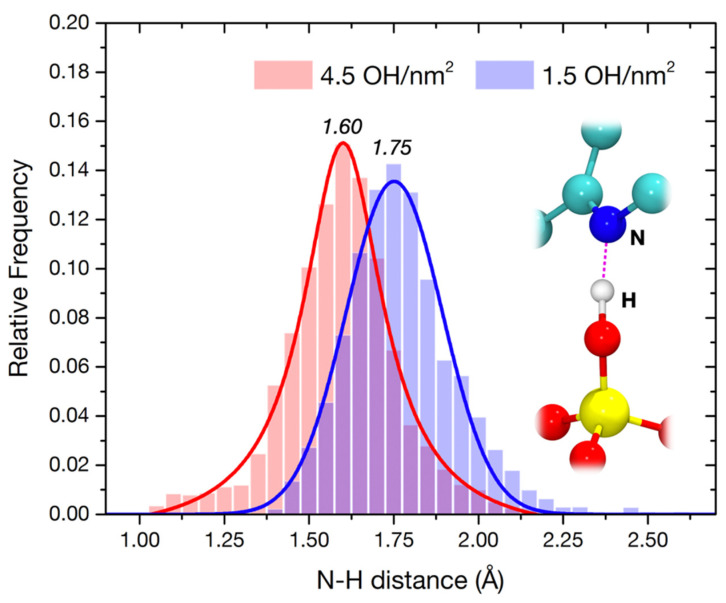
H-bond length distribution during the AIMD trajectories, for nitrazepam adsorbed on the 4.5 (red) and 1.5 (blue) OH/nm^2^ silica surfaces, between the diazepine N and the closest silanol group, as evidenced by the superimposed ball and stick model. Lines are Gaussian fittings to help the comparison.

This destabilization is clearly observed when analyzing in detail the dynamics of the bond between the diazepine N4 and the closest silanol group (Figure 5). The average H-bond length (1.75 Å), for the 1.5 OH/nm^2^ surface, seems to be not compatible with an impending proton transfer, while even exploring higher distances, corresponding to a very unstable interaction. This does not happen in the 4.5 OH/nm^2^ case. The distribution is narrower, in agreement with the lower RMSD of the atomic positions (Figure 4a). Furthermore, the AIMD allows the exploration of very short N–H distances, in some cases corresponding to a completed proton transfer, in agreement with the supposed catalytic pathway of degradation of Figure 1d. The fact that this process is indeed observed during a short unbiased AIMD at 300 K suggests a very low transition barrier, at least on a 4.5 OH/nm^2^ silica surface and when a particularly acidic silanol, i.e., terminal of a H-bonded chain, is involved. Since this transfer is seen multiple times in the simulation, we could indeed obtain a rough estimate of its characteristic time (*τ* ≈ 2 ps), corresponding to a rate constant k ≈ 5 × 10^11^ s^−1^ and a transition barrier at 300K ΔG^‡^ ≈ 6.3 kJ·mol^−1^. The protonated intermediate is, however, never stabilized in the simulation, quickly reconverting back to the neutral form of nitrazepam. In the real degradation process, this intermediate would probably be stabilized by solvating water, which we have recently predicted to be present even in a low humidity environment [52], to proceed towards the ring hydrolysis.

#### 3.3.4. IR Frequencies

**Figure 6:**
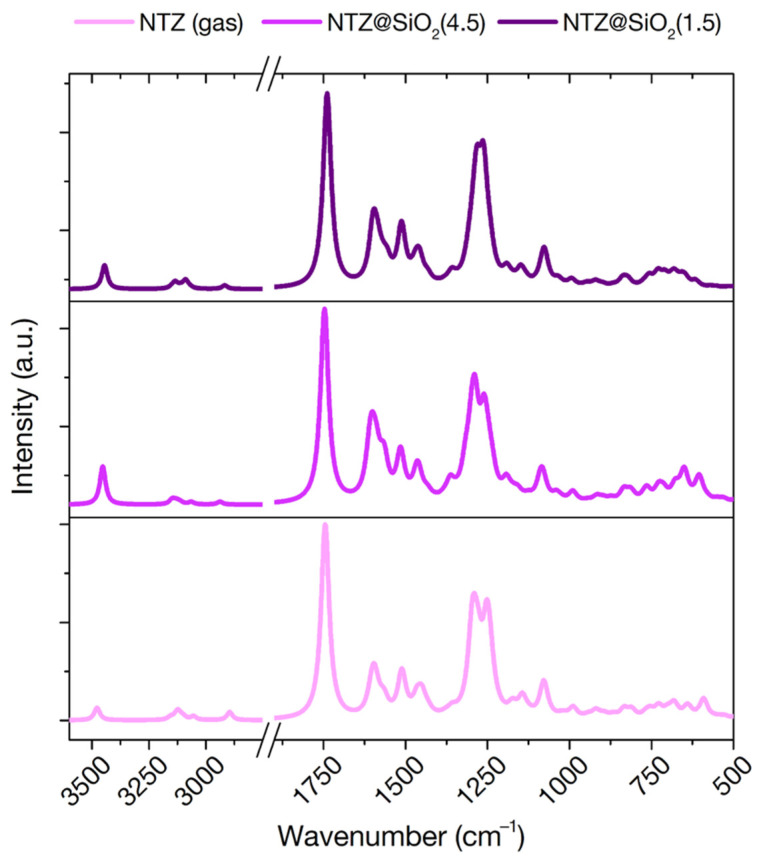
Simulated infrared spectra of nitrazepam in the gas phase (bottom) and adsorbed on the 4.5 (middle) and 1.5 (top) OH/nm^2^ silica surfaces. Intensities are scaled to highest peak for each spectrum.

FT-IR spectroscopy is a powerful experimental technique to investigate the chemical and physical state of molecules in interaction with inorganic surfaces. All the IR signals that fall in a range where the silica support is transparent are observable and can be traced back to the kind of interactions happening at the surface [8]. FT-IR spectroscopy has indeed been applied to several molecule/silica systems in the dry state [8,13,50]. The theoretical prediction of vibrational spectra can therefore act as a powerful link between experiment and simulation, providing both a validation of the models and a guide towards the interpretation of the measurements. We have indeed exploited this comparison in the past, for the cases of ibuprofen and clotrimazole in interaction with mesoporous silica particles [13,20]. Few published results exist however for the nitrazepam case. An experimental band assignment for the molecule in solid phase is reported [48,53], while a limited 1982 spectrum exists for nitrazepam in interaction with amorphous silica [30]. To guide future experiments, we report here the vibrational analysis of this drug molecule both in gas phase and in interaction with the two 4.5 and 1.5 OH/nm^2^ silica surfaces, using the PBE-D2 optimized adsorption geometries (Figure 6).

Unfortunately, limited band shifts are observed when moving from gas to adsorbed phase, suggesting that FT-IR might not be the best experimental techniques to investigate this interaction. Indeed, since only one functional group was predicted to interact with surface silanols, little perturbation should be expected for the other, FT-IR active, functionalities. Of particular interest is the ring C=N stretching vibration, which for benzodiazepine is known to occur in the regions 1615–1575 and 1520–1465 cm^−1^ [53]. We predict this band at 1607 cm^−1^ in the gas phase. This signal should be particularly sensible to destabilization of this bond due to an impending hydrolysis of the ring. However, only very limited shifts are predicted on both surfaces (1610 and 1601 cm^−1^ on the 4.5 and 1.5 OH/nm^2^ surfaces, respectively). This suggests that the N---H H-bond, even with an impending proton transfer, is not destabilized enough to give a discerning signal in FT-IR spectra. Interestingly, the signal that is most affected by adsorption is the one corresponding to the N-H group of the ring, which moves from 3478 cm^−1^ in the gas phase to 3453 cm^−1^ and 3445 cm^−1^ on the 4.5 and 1.5 OH/nm^2^ surfaces, respectively, despite not directly participating in H-bonds. This signal, however, is expected to be largely covered by the silanol stretching signals of the silica material, not acting as a valuable fingerprint of adsorption.

## 4. Conclusions

We have provided a static and dynamic characterization of nitrazepam in interaction with realistic amorphous silica surfaces, as a model for a 1,4-benzodiazepine encapsulated in mesoporous silica.

Our investigation proved that drug adsorption is strongly exothermic, driven by a balance of vdW interactions with the surface and strong H-bonds between the nitrogen atom of the Schiff base bond of the diazepine ring and exposed silanols.

The interaction is strong regardless of the hydroxylation level of the surface, but molecular dynamics proved that it is more stable on a hydrophilic surface with as silanol concentration of 4.5 OH/nm^2^. This stability is due to a very short H-bond with a silanol terminal of a long H-bonded chain. The known acidity of such surface moieties promotes a proton transfer to the Schiff base bond that could initiate bond hydrolysis and ring opening, according to the renowned path of drug degradation (Figure 1d), even in a microsolvating environment. Isolated silanols on a hydrophobic surface, with a silanol concentration of 1.5 OH/nm^2^, while strongly coordinating the nitrogen of the same bond, seem to be less prone to proton transfer.

Additionally, we provide FT-IR simulated spectra of the molecule both free and adsorbed at the surface that can be used as a guidance in the interpretation of experimental spectra. However, we predict these measurements to be non-conclusive regarding the adsorption geometry and a possible Schiff base bond destabilization induced by silica.

In conclusion, the atomistic picture offered by our quantum mechanical simulations envisages that mesoporous silicas are not a good candidate for nitrazepam drug delivery and of 1,4-benzodiazepines in general, due to a probable catalytic role of the surface towards drug degradation. This effect could be mitigated by thermal treatment of the material, reducing the density and acidity of surface silanols, while maintaining a strong drug–silica interaction.

## Figures and Tables

**Table 1:** Energetics of the nitrazepam/silica system. Computed electronic interaction energies, with (∆E^D^) and without (∆E) including dispersion (Disp.), corrected for BSSE. Results are given for structures optimized at both the PBE and PBE-D2 levels of theory. Values in parentheses refer to the PBE-D2 optimized structures after AIMD (cf. text for details). All values are in kJ·mol^−1^.

		∆E	∆E^D^	Disp.
4.5 OH/nm^2^	PBE	−30.8	–	–
	PBE-D2	−10.1	−104.9 (−128.0)	−94.8
1.5 OH/nm^2^	PBE	−38.7	–	–
	PBE-D2	−28.2	−131.9 (−100.2)	−103.7

## Data Availability

The data presented in this study are available on request from the corresponding author.
